# Probiotics in Postoperative Pain Management

**DOI:** 10.3390/jpm13121645

**Published:** 2023-11-25

**Authors:** Barbara Fyntanidou, Aikaterini Amaniti, Eleftheria Soulioti, Sofia-Chrysovalantou Zagalioti, Sofia Gkarmiri, Angeliki Chorti, Lamprini Loukipoudi, Aris Ioannidis, Ioannis Dalakakis, Alexandra-Eleftheria Menni, Anne D. Shrewsbury, Katerina Kotzampassi

**Affiliations:** 1 Department of Emergency Medicine, Aristotle University of Thessaloniki, 54636 Thessaloniki, Greece; fyntanidou@auth.gr (B.F.); sofia_zag@yahoo.com (S.-C.Z.); sofia.gkarmiri@gmail.com (S.G.); 2 Department of Anesthesia & Intensive Care, Aristotle University of Thessaloniki, 54636 Thessaloniki, Greece; aamaniti@auth.gr (A.A.); linaloukipoudi@gmail.com (L.L.); idalakakis@auth.gr (I.D.); 3 Second Department of Anesthesiology, National and Kapodistrian University of Athens, Attikon University Hospital, 12462 Athens, Greece; eleftheriasoulioti@gmail.com; 4 Department of Surgery, Aristotle University of Thessaloniki, 54636 Thessaloniki, Greece; chorange@auth.gr (A.C.); ariioann@yahoo.gr (A.I.); alexmenn@auth.gr (A.-E.M.); a_shrewsbury@yahoo.com (A.D.S.)

**Keywords:** probiotics, postoperative pain, microbiome, visceral pain, abdominal wall pain, inflammation, opioids, cannabinoids

## Abstract

Postoperative pain is the unpleasant sensory and emotional experience after surgery, its origin being both the inflammatory reaction induced by the surgical trauma on the abdominal wall and the splanchnic pain induced by the activation of nociceptors of the viscera, which are highly sensitive to distension, ischemia, and inflammation. Nowadays, it is well recognized that there is a close relationship between the gut microbiome and pain perception, and that microbiome is highly affected by both anesthesia and surgical manipulation. Thus, efforts to restore the disturbed microbiome via supplementation with beneficial bacteria, namely probiotics, seem to be effective. In this article, the knowledge gained mainly from experimental research on this topic is analyzed, the concluding message being that each probiotic strain works in its own way towards pain relief.

## 1. Introduction

The word “pain” is thought to have come from the word “ponos,” the modern Greek word for pain, which originally meant labor and hard work in the Homeric epics. This meaning may have originated from the personification of “ponos” (toil) and “algea” (sorrows) as children of the hard-hearted goddess “Eris” (Strife) in Greek mythology. (Ἔρις στυγερὴ τέκε μὲν Πόνον ἀλγινόεντα,Λήθην τε Λιμόν τε καὶ Ἄλγεα δακρυόεντα) (Hesiodus, Theogonia, 0020.001) [1]. Ponos is the name of the Greek goddess of revenge, sent to punish mortal fools who had angered the Olympian gods because they had accepted and used the fire stolen by Prometheus from Mount Olympus. A similar belief about pain as the revenge of a goddess was also common in many ancient cultures. Similarly, in the Bible, when Adam and Eva were banished from Eden, God’s punishment was for them to experience pain and for Eva to be subjected to the severe intermittent pain of birth (“odynes”).

Aulus Cornelius Celsus (25 BC-50 AD), in his famous medical work *De Medicina*, was the first to record the cardinal signs of inflammation known as the “Celsus tetrad of inflammation”: calor (warmth), dolor (pain), tumor (swelling), and rubor (redness and hyperemia). He also went into great detail regarding the preparation of numerous ancient medicinal remedies including opioids, also greatly valued by Hippocrates (460–370 BC) for the treatment of pain, most frequently recognized as “algos” (άλγος), “algema” (άλγημα), “odyne” (οδύνη), and “ponos” (πóνοσ) [2].

The Greeks and Romans were the first to inspire the “theory of sensation”, that is, the theory that the brain and nervous system intervene in the perception of pain. But it was not until the Middle Ages, and well into the Renaissance—the 1400s and 1500s—that evidence began to accumulate to support these theories. Leonardo da Vinci and his contemporaries came to believe that the brain is the central organ responsible for sensation and that the spinal cord transmits sensations to the brain [2].

Today, the International Association for the Study of Pain has updated the definition of pain to “an unpleasant sensory and emotional experience associated with, or resembling that associated with, actual or potential tissue damage” [3]. However, in parallel with the ongoing knowledge of the interference by the body’s overall microbiome in almost any disease pathophysiology, there is an augmented literature on the implication of the gut microbiome in pain perception. The close association of the gut microbiome, and more specifically of the low-diversity microbiome or pathobiome, with functional bowel diseases in the form of “chicken and egg” has been well recognized for years. Only in recent decades has the cluster of beneficial bacteria belonging to the microbiome, i.e., probiotics, begun to be recognized as implicated in the manipulation of the damaged microbiome, to serve as an “alternative” treatment of high effectiveness [3].

The purpose of this narrated review is to present the current knowledge, mainly derived from animal experiments, in relation to postoperative pain management, by means of specific probiotic strains. For an overall understanding of the true implication of the microbiome in pain perception and vice versa, we first look at the known association between the microbiome and pain and then to the alterations to the gut microbiome as a consequence of anesthesia and surgery.

## 2. Pain and the Gut Microbiome

The gut microbiota typically hosts thousands of bacterial species, as well as human viruses, fungi, and bacteriophages [4,5,6]. This intricate ecosystem, which is continually reshaped by the host and its environment, and, at the same time, affecting its function and health, is now defined as a “microbiome”, the term including both the microbiota within its well-defined habitat and all the bacterial and external structural parts, including their genes [7,8].

Microbiota generally live in a significant relationship, called homeostasis, with the host gut, the interactions regulated by the integral gut barrier and the immune system [9,10]. On the other hand, the gut bidirectionally communicates with the central nervous system via immunological, hormonal, and neural pathways, the complex interaction being named the gut–brain axis [11]. Today, when everyone recognizes the microbiome as an integral part of the gut, both in the mucosa and the lumen, and as influencing the bidirectional signaling pathways between the central nervous system and the gut, this gut–brain axis is called the microbiome–gut–brain axis [12,13,14,15,16]. 

**Figure 1:**
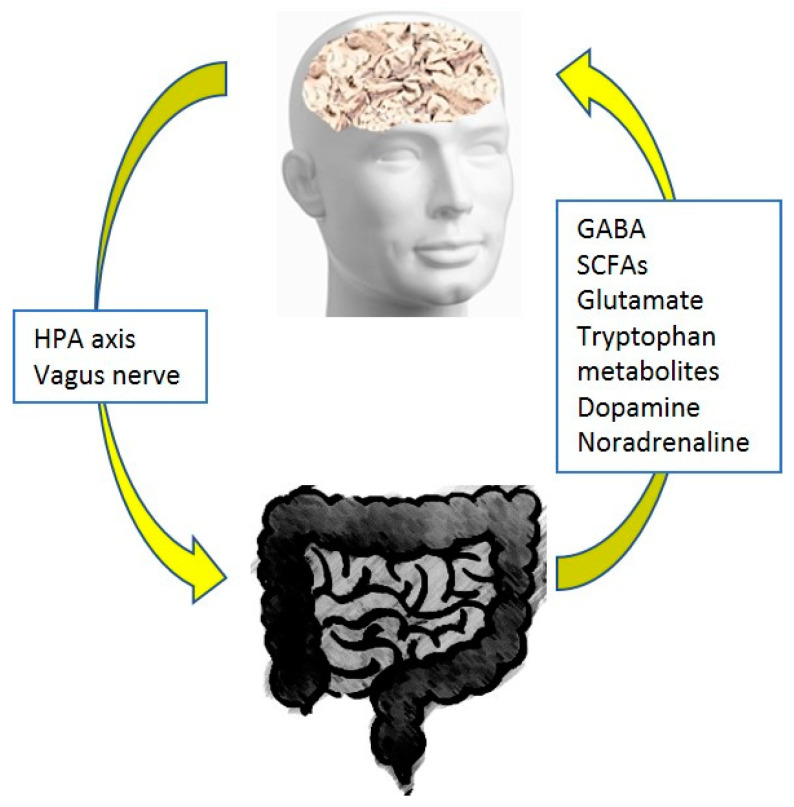
The microbiome–gut–brain axis.

In this context, the gut microbiome can synthesize and secrete various neurotransmitters and neuro-modulatory bacterial metabolic end products, including short chain fatty acids (SCFAs), tryptophan metabolites, gamma aminobutyric acid (GABA), glutamate, dopamine, and noradrenaline [17,18,19,20,21]. In physiological conditions, the blood–brain barrier permits the passage of tryptophan metabolites and SCFAs into the central nervous system, but does not allow the circulating neurotransmitters, excluding GABA [21,22,23,24]. Disruption of the blood–brain barrier, due to any pathology, permits an increased number of circulating neurotransmitters to enter the brain. Injection of butyrate, the main SCFA produced from certain microbial strains, into rat and mouse brains was revealed to stimulate the release of the brain-derived neurotrophic factor favoring neurogenesis. Additionally, butyrate is considered to exert regulatory effects on inflammation-induced visceral pain [24,25]. See Figure 1.

Bacteria such as *Prevotella*, *Fusobacterium*, *Enterococcus casseliflavus*, *Escherichia*, and *Bacteroides* were found to produce tryptophan, which, by passing the blood–brain barrier, allows serotoninergic neurotransmission in the brain [26]. Glutamate, produced by certain microbial strains, is also a major neurotransmitter in the central nervous system which also regulates gut sensory and motor functions in the enteral nervous system [27,28,29,30]. In cases of dysbiosis due to stress, the expression of glutamate receptors is altered, while in dysbiosis due to antibiotic treatment in mice, the decreased levels of hippocampal N-methyl-D-aspartate receptors and brain-derived neurotrophic factors were able to be restored by probiotic/prebiotic treatment [31,32,33,34].

Early studies associated the altered structure of the gut microbiome (decreases in *Bifidobacterium*, *Faecalibacterium*, and *Faecalibacterium prausnitzii*, and increases in *Lactobacillaceae*, *Bacteroides*, and *Enterobacteriaceae*) with human digestive tract diseases [35,36]. Nowadays, there is augmented evidence that the gut microbiota is one of the most important pain modulators, working towards pain regulation in both the central and peripheral nervous systems. This evidence is much more pronounced in cases of visceral or abdominal pain [37,38], such as irritable bowel syndrome, for which we know that the diversity of the microbiome is not only directly related to the diagnosis, but also to its phenotypic subtypes, i.e., predominantly diarrhea or constipation, as well as to the severity of the syndrome and its flare-ups [39,40,41]. There are also significant indications for inflammatory pain, migraine, joint pain, neuropathic pain, and opioid tolerance [15].

Today, it is well accepted that there exists a complex bidirectional signaling network between the brain and the enteric bacteria through various neural (vagus, enteric neural system, and spine nerves), neurotransmitter (GABA, noradrenaline, serotonin, dopamine, and glutamate), and molecular signaling pathways. The noxious stimuli activate the primary nociceptive neurons located in the trigeminal and dorsal root ganglion (DRG), and these primary afferent signals convey the nociceptive stimuli to the gray matter of the dorsal horn in the spinal cord. In parallel, metabolites produced by the gut bacteria indirectly stimulate the enteric sensory neurons to transfer the “information” directly to the brain by means of the vagus nerve (parasympathetic nervous system). The detailed discussion of this complex phenomenon is beyond the scope of this article. Thus, we refer to the very recent and excellent publication by Morreale C et al. [42], as well as the articles by Brenner D et al. [43] and Chen O et al. [44].

The fundamental role of the gut microbiota has been confirmed in mice that had undergone spared nerve injury, who then received fecal transplantation from pain-free donors or from patients suffering chronic postoperative pain. Mechanical thresholds were found significantly decreased after receiving feces from pain-suffering patients rather than from pain-free donors, and this was accompanied by a significant reduction in the levels of the peroxisome proliferator-activated receptor γ (PPAR γ), a key metabolism modulator implicated in pain and a trend towards an increase in activated microglia in the spinal cord [35,45,46,47].

In another set of experiments in germ-free mice subjected to systemic LPS or ischemia/reperfusion injury, Amaral et al. found that the lack of microbiota was followed by inflammatory hypo-responsiveness, mediated by IL-10 via inhibition of the release of inflammatory cytokines and of the expression of COX-2, since IL-10 down-modulates hyper-nociception, which is a state of increased sensitivity to painful stimuli, or, in other words, a decrease in the behavioral nociceptive threshold [48]. Microbiota transplantation thereafter was followed by loss of the ability to produce IL-10 and a regained ability to develop inflammation in response to diverse stimulations. All of the above imply a critical role in the interaction between the host and the commensal bacteria in favoring adaptation to environmental stresses, including those which release pain.

Microbiome analysis of fecal samples from patients suffering from chronic pelvic pain syndrome and healthy controls was correlated with the NIH-Chronic Prostatitis Symptom Index. Patients have significantly decreased α-diversity and a wider clustering in relation to controls, who revealed tighter clustering in a space distinct from the patients. Additionally, compared with the controls, 3 taxa were over-represented and 12 were under-represented, as occurs with *Prevotella* [49].

In a recent clinical study, gut microbiota diversity and abundance were analyzed in 20 patients undergoing surgical fixation of distal radius fractures under an axillary brachial plexus block. The results were then correlated with (i) the verbal pain rating scale; (ii) the level of pain deemed “acceptable” by the patient; (iii) the 24 h max self-reported pain score; and (iv) the 7-day analgesic consumption. The major finding was that the 7-day analgesic consumption was inversely correlated with the Shannon index of α-diversity, known to be decreased in certain pain conditions [50]. Pain perception was found to be associated with the abundance of certain microbial genera, including *Collinsella*, previously recognized to influence the production of the proinflammatory cytokine IL-17A, to increase gut permeability and disease severity in experimental arthritis [51]. In patients whose pain was perceived as “not acceptable”, a greater abundance of *Lachnospira* and *Alistipes* was prominent, while the analgesic consumption was positively correlated with abundance of *Dialister*, previously shown to correlate with ankylosing spondylitis [52].

Finally, in an acute pain rat model, *Porphyromonas gingivalis* LPS locally applied resulted in acceptable levels of pain, the antinociceptive action being mediated by a significant increase in the anti-inflammatory cytokine IL-10 levels [53], while the lack of *Akkermansia muciniphila* in the feces of patients suffering from irritable bowel syndrome seems to be inversely correlated with pain [54].

## 3. Surgical Operation and the Microbiome

**Figure 2:**
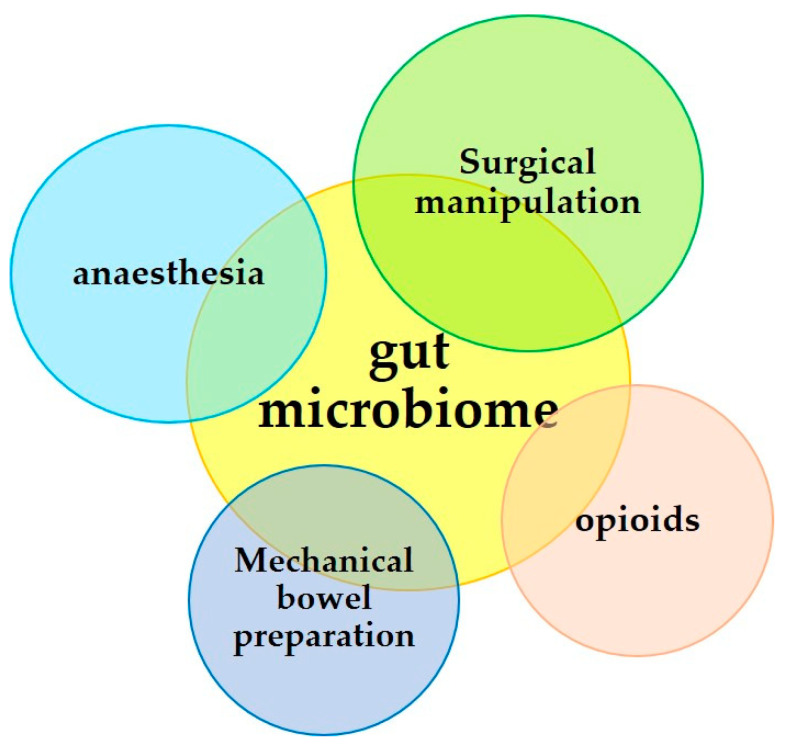
Parameters of the surgical procedure affecting the gut microbiota.

For the needs of the present analysis, the term “surgical operation” includes all those manipulations that precede the main surgical time of a programmed laparotomy, i.e., the procedure of preoperative mechanical bowel cleansing, when required, and the administration of anesthesia/analgesia. However, in cases of emergency surgery, the medical event leading to the operating theater must also be taken into account—severe trauma, gut perforation, or gut obstruction being the most common—as seriously damaging the body and its microbiome. The possibility of preoperative food deprivation as well as the administration of antibiotics and, perhaps, opioids for pain management must not be overlooked [55,56,57,58,59,60]. See Figure 2.

Mechanical bowel preparation aimed at reducing the amount of fecal material and bacterial count with the objective of decreasing surgical site infection rate, including anastomosis leakage, has been a common practice for more than a century. However, cleansing preparations osmotically increase the volume of water within the gut, washing out the lumenal contents, including gut bacteria. Additionally, the rapid increase in bowel movements flushes out bacteria incapable of adhering to the gut mucosa, distorting the fecal microbiota composition [61,62,63,64].

By approaching the issue of bowel cleansing from the perspective of the gut microbiome, an early randomized controlled trial reports a significant decrease in the total bacteria numbers of the *Clostridium coccoides* group, the *Clostridium leptum* subgroup, *Bifidobacteria*, *Lactobacillus*, and *Enterobacteriaceae*, but no effect on *Enterococci* and *Staphylococci* [65]. Jalanka et al., in feces samples collected immediately after bowel cleansing, reported a reduction in the number of bacteria in relation to the baseline, and the number of methanogenic archaea per gram of feces was decreased by 20-fold [61]. Specifically, they found a decrease in the members of *Clostridium cluster IV*, and increases in the members of *Clostridium cluster XIVa* and *Proteobacteria*, *Fusobacteria*, and bacteria related to *Dorea formicigenerans*, with all returning to the baseline only after the 14th and 28th days. More recent studies have documented these findings: At the phylum level, mechanical cleansing resulted in a reduction in the relative abundance of *Firmicutes* and an increase in *Proteobacteria*, both restored one month later; and at the class level, it resulted in increases in *γ-Proteobacteria* and *Coriobacteria*, and a significant reduction in *Clostridia*. Finally, at the family level, a significant reduction in *Lactobacilli* and a rise in *Enterobacteriaceae* were found to have persisted one month later [66,67].

Regarding the procedure of anesthesia, it is now recognized that it could provoke perioperative neurocognitive dysfunction, attributable to modifications in the gut microbiome. The use of certain general anesthetics could result in a depletion of microbial α-diversity, and of neurologically relevant metabolite variations. Most of our knowledge on the association between anesthetics and microbiota has been derived from studies testing different anesthetics to inhibit infections after surgery [68], their findings supporting a wide range of effects, including phenotype modifications, the changes seeming to depend mostly on the duration of anesthesia [69,70]. Isoflurane and sevoflurane given in vitro, but not propofol, were found to increase the motility and biofilm formation of pathogenic *E. coli*, *S. aureus*, *E. faecalis*, and *P. aeruginosa*, both changes being associated with increased bacterial virulence but also being drug-dependent [71]. Moreover, Maier et al. have evaluated the effects of different drugs, including propofol and etomidate, on the growth of the 40 most common bacteria in the human gut. Etomidate, but not propofol, showed significant antibacterial activity against *Ruminococcus bromii*, *Roseburia intestinalis*, *Ruminococcus torques*, and *Blautia obeum* [72].

The association between anesthetics and gut microbiota is now clarified by their shared affinity for neurotransmitters, which empower the anesthetics to directly interact with prokaryotes. Additionally, acute variations in the levels of neurotransmitters due to anesthesia can lead to transitory changes in the gut mucosa, including postoperative gastrointestinal tract dysfunctions, commonly observed in abdominal surgery patients [73,74,75]. The impact of anesthesia on the gut milieu has also been considered an essential trigger of neuroinflammation and the development of cognitive dysfunction [76,77,78,79].

Recent experimental models have demonstrated a significant alteration in the gut microbiome after exposure to anesthetics, even after a short-term exposure. Moreover, these studies suggest bidirectional interactions: alterations in the composition of the microbiota after anesthesia, and effects of the altered microbiome on postoperative outcomes, including postoperative pain and postoperative cognitive dysfunction. Both of these are prominent in early studies in humans, showing a close relationship between microbiota composition and postoperative outcomes, including pain and delirium [35]. Specifically, Han et al. showed a strong perturbation in the fecal microbiome following sevoflurane anesthesia in mice, the changes lasting for more than 14 days: the genera *Bacteroides*, *Alloprevotella*, and *Akkermansia* remained significantly elevated in relation to the baseline and did not return to their original levels [80]. Similarly, the genus *Lactobacillus* remained significantly reduced even after two weeks. In the same manner, propofol given intravenously for 3 h in rats induced gut microbiota changes: reductions in *Prevotella*, *Alloprevotella*, and *Lactobacillus*, showing a trend to recover two weeks later [81].

Furthermore, Liufu et al. found in mice that a 1.4% isoflurane anesthesia of two hours duration, in conjunction with a ten-minute surgery (extended laparotomy and immediate closure)—which potentiates the anesthesia’s neurotoxicity—could cause changes in gut microbiota, changes being age-dependent [82,83,84,85]. Specifically, significant alterations in the composition of the gut microbiota were found in older mice (18 months), but not in 9-month-old mice. However, there were different levels of microbiota diversity in the two age groups, the reduction rate in the abundance of *Lactobacillus* at genus and species levels being, generally, more pronounced in the older mice, especially in the case of *Lactobacillus salivarius*, which also had a longer recovery time of more than 11 days. However, it should be noted that a 2.5% lidocaine plus 2.5% prilocaine local anesthetic cream was applied to the incision site three times daily up to euthanasia, to alleviate pain and thus avoid the interference of pain in microbiome alterations [83,84,86].

Regarding the consequences of surgical manipulation on the gut microbiome, it has long been accepted that operational stress can induce alterations in the intestinal bacteria via corticotropin-mediated routes, which can activate systemic inflammatory responses [55,87,88,89]. Surgery represents a form of controlled trauma, which is an established source of tissue injury, and every injury is a key driver of inflammation [90]; the cellular damage triggers endogenous factors, the damage-associated molecular patterns, which activate immune cells to resolve the damage and restore homeostasis [91,92]. However, the physical trauma of surgery itself, in cooperation with the surgery-induced alterations in tissue oxygenation, perfusion, and pH and augmented by the stress of anesthesia and the intraoperative pharmacological manipulations, has long been shown to cause gut microbial dysbiosis [93,94]. In colorectal surgery patients, when comparing pre- and postoperative stool samples, a significant decrease in total bacterial counts and in the numbers in six groups of obligate anaerobes was prominent [95], while the populations of *Enterobacteriaceae*, *Enterococcus*, *Staphylococcus*, and *Pseudomonas* were significantly increased [96].

A systematic review of 10 studies scoping the relationship between the gut microbiome and postoperative complications has suggested that complications might be influenced by the patients’ gut microbiota [97]. Surgical procedures of different types tend to result in an increase in potentially pathogenic bacteria such as *Pseudomonas*, *Staphylococcus*, and *Enterococci* and a decrease in *Lactobacilli* and *Bifidobacteria* [98,99,100].

An early study by Shogan et al. in rats subjected to colon resection and anastomosis, as is usually performed in colon cancer patients, revealed that colorectal surgery does not affect the bacteria of the lumenal contents (stool), but induces significant alterations in the composition of those that are adherent to the gut epithelium [101]. Most of the changes were observed in *Proteobacteria*, *Actinobacteria*, and *Firmicutes:* among *Proteobacteria*, the relative abundance of *Escherichia/Shigella* increased 200-fold; in Actinobacteria, the relative abundance of an uncultured bacterium increased approximately 50-fold; and nonuniform changes among the Firmicutes genera were also prominent. On the other hand, the relative abundance of *Ruminococcaceae* and *Clostridia* decreased 20-fold, that of *Allobaculum* and *Coprococcus* increased 10-fold, and that of Enterococcus increased 500-fold. Finally, a 20-fold decrease in *Prevotellaceae* clearly suggests that Bacteroidetes were also affected by anastomotic injury [101].

In a recent review, Agnes et al. questioned “How surgery affects the gut microbiome?” in order to analyze the different factors involved. Besides the operational stress and the disruption of tissue homeostasis, the authors considered that many species of gut microbiota, being either facultative or obligate anaerobes, when exposed to oxygen during colon surgery, could be subjected to a significant effect on their species; in other words, a reduction in “good” obligate anaerobes, such as some *Bacteroides*, and an increase in “bad” facultative anaerobes, such as *Enterococcus*, could occur [89,101]. Another cause of decrease could also be tissue ischemia or hypoperfusion, due to permanent or temporary occlusion of gut vessels. In an ischemia/reperfusion model in rats, 30 min of gut ischemia followed by reperfusion resulted in changes in the gut microbiota at 1 h of reperfusion, reaching the peak at 6 h. This was followed by progressive recovery of *E. coli* and *Prevotella* first and *Lactobacillus* later [102,103,104].

However, the extent of surgery-induced gut microbiome depletion is also related to or affected by different factors, such as the length of the surgical line incision or the type of suture used [82,105]. Although there are no references as to the exact relationship between the gut microbiome and the degree of inflammatory reaction caused by surgery, it is clear that less abdominal wall trauma occurs in laparoscopy versus laparotomy and/or less inflammation occurs when using absorbable sutures or other implanted material, which should result in less aseptic inflammation and thus less pain and less catabolism [106]. The catabolic modifications that develop mainly in muscles to provide energy and essential amino acids to improve repair of damaged tissues and wound healing and to maintain the function of the critical organs must not be overlooked [55,107,108]. However, catabolic hormones such as catecholamines and cortisol are known to increase, proportional to the extent of trauma of any type and etiology. It is also known that at least noradrenaline can interact with *Escherichia coli* and the *Pseudomonas aeruginosa* quorum sensing receptors and activate the transcription of some microbial genes to virulent phenotypes [68,109]. The same cell-to-cell signaling mechanism that allows the release and detection of extracellular signals, called quorum sensing, has also been recognized after 5-HT, substance P, or epinephrine release, which all increase the motility of the virulent phenotype of *Pseudomonas fluorescens* [110,111].

Finally, of importance is the bidirectional interaction among opioids, opioid receptors, and the microbiome: opioids were found to modulate the diversity of the gut microbiome, and that, in turn, affects the host response to opioids. Morphine given to mice resulted in significant alterations in the gut microbiome, evident as early as 1 day after administration, but were reversible via the coadministration of the μ-receptor antagonist naltrexone [112]. More interestingly, the host response to morphine was dependent on its microbiome: in an experimental model of morphine tolerance, naive mice were more prone to developing tolerance than germ-free and antibiotic-treated mice [113]. Moreover, the tolerance was found to be related to the decrease in *Bifidobacteria* and *Lactobacillaeae*, which, when they “re-enter” the gut by means of fecal transplantation, re-establish opioid tolerance in this model [35,68].

## 4. Probiotics in Postoperative Pain Management

Based on the above, we may say that the effort to control or reduce postoperative pain, by any means, has two simultaneous approaches, one relating to the pain of the surgical trauma on the abdominal wall which is purely of an inflammatory etiology, and the other to the visceral pain, which is due to overdistension of the intestine before its mobilization from the paralytic ileus, resulting from anesthesia.

According to the current definition, “probiotics are live microorganisms that, when administered in adequate amounts, confer a health effect on the host” [114]. Today, many in vitro and in vivo studies have shown that probiotics exert potential suppressive effects on various infectious and inflammatory conditions, thus significantly shortening the inflammatory phase of the wound healing process [115,116,117]. At the same time, there are quite a lot of clinical studies referring to the reduction in postoperative bloating and the shortening of the time duration up to the first flatus, all indirectly indicating the shortening of the postoperative paralytic ileus [118,119,120]. Both the above [115,116,117,118,119,120] may not have a direct, suppressive effect on the pain originating from abdominal wall trauma, or from the distension of the gut wall, but there are many studies documenting the specific cytokine and other molecular changes which point in that direction.

### 4.1. Probiotics in Relation to the Inflammation-Induced Pain of Surgical Trauma

Surgical incisional trauma causes inflammatory reactions as part of the healing process. Inflammation seems to contribute to the sensitization of peripheral nociceptors, leading to hyperalgesia—a situation in which people have higher sensitivity to pain, or allodynia, when noxious or non-noxious stimuli cause pain [121]. Inflammation reduces the pain threshold of nociceptors and increases the individual pain response. Postoperative pain is exacerbated by the released proinflammatory cytokines, not only locally but in the spinal dorsal horn tissues, which are further involved in the pain mechanism, the magnitude of pain being proportionally dependent on the incision length and the surgical manipulations [105,122,123]. The released chemo- and cytokines trigger the activation of the intracellular downstream signal pathways and, subsequently, the phosphorylation of receptors and ion channels in primary sensory neurons. This whole process leads to neuronal hyperexcitability and peripheral sensitization [15]. Furthermore, it is known that skin-deep incisions induce mechanical and heat hypersensitivity similar to that of incisions extended deeper than the skin; thus, cutaneous trauma drives the majority of postoperative pain due to the major inflammatory process, or the opposite: a deep trauma elicits major inflammation and thus much pain [124,125,126].

Probiotic species seem capable of modulating the gut microbiota to prevent or alleviate inflammatory pain, the beneficial effects being the consequence of their multifaceted action: controlling proinflammatory signaling via increased expression of anti-inflammatory cytokines, or via directly limiting the bioavailability of specific proinflammatory cytokines [115,127]. The anti-inflammatory IL-10 binds to the IL-10 receptor, with the IL-10R1 being the high-affinity subunit necessary for signal transduction, expressed by astrocytes, microglia, endothelial cells, and trafficking leukocytes [128]. Its anti-inflammatory action is accelerated by blocking downstream signaling of TLR4, which normally promotes a proinflammatory milieu, critical for the induction of neuropathic pain [129]. Additionally, IL-10 signaling also induces the production of the suppressors of cytokine signaling 1 and 3, thereby further reducing proinflammatory cytokine production by targeting the p65 NF-κB subunit [130]. This effect has also been confirmed in a clinical study on colorectal surgery patients treated with the four-probiotic regime of *L. plantarum*, *L. acidophilus LA-5*, *B. lactis BB-12*, and *S. boulardii* [118].

Diabetic rats subjected to a dorsal wound and topically treated with *Lactiplantibacillus plantarum* presented a significantly increased expression of the anti-inflammatory mediators IL-10 and TGF1 up to day 14, in parallel with a markedly decreased expression of the proinflammatory IL-1 and TNF on day 7 and thereafter, in comparison with control-treated rats. These findings were consistent with the speeding up of wound closure on day 3 and thereafter [131]. *Staphylococcus aureus*-infected excisional wounds treated with *L. plantarum MTCC 2621* exhibited upregulation of IL-10 in the later phase of the healing process in relation to controls, in parallel with earlier re-epithelization, accompanied by a reduction in leukocyte infiltration and increased activity of fibroblasts and deposition of collagen [132]. Other probiotic bacteria, such as *L. rhamnosus UBLR-58*, *L. acidophilus LA-5*, *L. fermentum SGL10*, *L. brevis GQ4237768*, *L. brevis SGL 12*, *L. paracasei SGL 04*, and *B. longum UBBL-64* also exert anti-inflammatory action by means of similar mechanisms, but to a significantly lesser degree, in relation to *L. plantarum* [133,134,135].

TNF is considered of particular value as a marker of inflammation due to its close relationship with the prostaglandin release implicated in pain, swelling, and fever [136]. In an ex vivo human skin explant model, the administration of the postbiotic *B. longum reuter* lysate led to an improvement in parameters relating to inflammation, but it mainly significantly inhibited capsaicin-induced CGRP release by neurons. Similarly, the same regime in humans led to increased skin resistance to physical and chemical aggression in relation to a control [137].

Contrary to these positive findings, *Lactobacillus reuteri LR06* and *Bifidobacterium longum BL5b*, given in the drinking water in a rat model of inflammatory pain by means of injection of complete Freund’s adjuvant into the left hind paw, were found to have no antinociceptive effect. This was assessed by means of mechanical and thermal hyperalgesia, while Iba1 was used to verify the microglial inflammatory activation in relation to a placebo [138].

There is also evidence that probiotic beneficial bacteria may affect inflammatory cytokine levels by acting on the NF-κB and mitogen-activated protein kinase (MAPK) signaling pathways [139]. *L. rhamnosus GG ATCC 53103* was found to modulate signal transduction pathways by triggering MAPKs, while *L. acidophilus LA-5* was found to show higher values in all MAPKs analyzed [140,141].

Finally, besides anti-inflammatory cytokines, microbiota also produce neurotransmitters, which are able to alter pain signaling. *Lactobacillus* spp., *B. dentium*, and *Bifidobacterium* spp. produce GABA, the most important inhibitory neurotransmitter in the brain, through enzymatic decarboxylation of glutamate.The authors suggest that GABA binds to its receptors on the DRG neurons, resulting in their depolarization, thus inhibiting the peripheral nociceptive transmission [142].

*Lactobacillus paracasei MSMC39*, given as a mouthwash to 30 patients who had had an impacted mandibular third molar removed, was found to significantly reduce the TNF level in gingival crevicular fluid in relation to a placebo. Pain, swelling, and trismus, evaluated using a visual analog scale score, were also reduced, but not to a statistically significant degree [143]. Thirty-eight patients subjected to third molar surgery were randomized to a *Levilactobacillus brevis CECT7480* and *Lactoplantibacillus plantarum CECT7481* or control regime for one week. Probiotics were found to significantly reduce pain and eating difficulty scores on the 5th postoperative day and thereafter. The infection rate and swelling values showed no significant difference between probiotics and the placebo at any time point [144].

A total of 283 patients with a single rib fracture were allocated to a regime of either *Lactobacillus casei Shirota* in skimmed milk or a placebo daily for a month following the fracture. Pain relief was assessed during activities specifically chosen to elicit pain, while sleep quality and maximal inspiration lung volumes were also examined. Probiotic treatment was found to be more effective in relieving pain during deep breathing, coughing, and turning over, while patients also had increased inspiration volumes, with sleep quality being unaffected [145] (Table 1).

### 4.2. Probiotics in Relation to Gut Distension-Induced Visceral Pain

#### 4.2.1. Experimental Studies

Visceral or splanchnic pain results from the activation of nociceptors of the thoracic, pelvic, or abdominal organs. Splanchnic organs are highly sensitive to distension, ischemia, and inflammation, but are relatively insensitive to other stimuli that normally evoke pain, such as cutting or burning [16,38]. Thus, the colorectal distension model—although scheduled for inflammatory bowel disease or irritable bowel syndrome simulation—could be considered a model characterized by visceral pain associated with altered transit time, as occurs in the postoperative situation of paralytic ileus, before the gut starts to move again.

One of the basic findings correlating probiotic strains with pain relief is that documented by Rousseaux et al. [146]. They initially hypothesize that some probiotics may stimulate the expression of receptors on epithelial cells that locally control the transmission of nociceptive stimuli to the gut nervous system. After testing different probiotic bacteria, they found that both *L. acidophillus NCFM* and *L. salivarius Ls-33* induced a sustained increase in opioid receptor μ- (OPRM1) mRNA expression in human HT-29 epithelial cells, but only the former, either live or heat-killed, was able to also induce significant cannabinoid receptor (CNR2) mRNA expression. Then, they conducted the same experiment on mice and rats, orally administering a live *L. acidophillus NCFM* strain for 15 days, and found the same receptors expressed in 25% to 60% of epithelial cells as compared with 0% to 20% in placebo-treated animals. Additionally, in treated rats, the mean colorectal distension required to induce pain was 20% more than the 50 ± 2 mm Hg in controls [146].

On the same side, live and heat-killed *Akkermansia muciniphila*, as well as their outer membrane vesicles and their cell-free supernatant (postbiotics) were used to infect Caco-2 and Hep-G2 cell lines. Quantitative real-time PCR revealed that all the forms of *A. muciniphila* were involved in the endocannabinoid system by means of affecting the cannabinoid receptor 1 and 2 expression; this was dose- and cell-line-dependent [47].

In a colorectal distension model, a commercially available fermented dairy product containing *Bifidobacterium lactis CNCM I-2494* and *Lactococcus lactis CNCM I-1631* and two common yogurt starters, *Streptococcus thermophilus* and *Lactobacillus bulgaricus*, were found to reduce stress-induced visceral hypersensitivity and pain. Of interest, the antinociceptive effect of fermented dairy products was dose-dependent and generally stronger than that of the *B. lactis CNCM I-2494* strain alone at the same dose, which are findings that support a synergistic interplay. In detail, the antinociceptive action of *B. lactis CNCM I-2494*, when given in a single dose per day, was clear at a dose of 10^10^ CFU while, when in coadministration with the fermented dairy product, it was clear at the lower dose of 10^8^ CFU [147].

In a model of colonic visceral hypersensitivity induced by infusion of 2,4,6-trinitro-benzene–sulfonic acid into the proximal colon, an 8-day probiotic *Lactobacillus rhamnosus Lcr35* treatment produces anti-hypersensitivity activity. As this model is known to involve an increase in IL-13 secretion, it may be that the action of probiotic treatment involves the regulation of the local IL-13/Th17 immune activation [148].

Colonic visceral hypersensitivity of inflammatory origin, occurring in rats after intracolonic instillation of zymosan, was found to be significantly attenuated by *Lactobacillus rhamnosus GG ATCC53103*, the visceromotor response for grading colorectal distension being determined via measurement of the electromyographic activity of the abdominal external oblique muscles. The levels of the neurotransmitters serotonin, noradrenaline, and dopamine and biogenic amines quantified in the frontal cortex, subcortex, brain stem, and cerebellum were also found to be significantly altered in *L. rhamnosus*-treated rats, suggesting that they could be involved in pain modulation [149,150].

Rats exposed to maternal separation, from weaning onwards, were given drinking water with or without supplementation with *Lactobacillus rhamnosus GG* soluble mediators. Maternal separation followed by restraint stress in adulthood led to increased splanchnic sensitivity and corticosterone plasma levels, as well as to alterations in β-diversity and abundance of specific bacteria, including *parabacteroides*—all these effects being ameliorated through *L. rhamnosus GG* soluble mediator supplementation [151].

Rats subjected to stress but having received placebo treatment exhibited a reduced representation of the pathways involved in the metabolism of butyrate and a reduced abundance of several operational taxonomic units associated with butyrate-producing bacteria, such as *Lachnospiraceae*. However, *Roseburia hominis* treatment led to alleviation of visceral pain perception and hypersensitivity, as well as to an increase in cecal butyrate concentration [152].

On the other hand, in a rat model of colonic hypersensitivity elicited via butyrate enemas, the hypersensitivity improved after administration of a *Lactobacillus acidophilus NCFM* strain, since treatment increases the colorectal distension threshold by 44% compared with that of untreated rats. It is very important to underline the fact that the antinociceptive effect of *L. acidophillus* was similar to that elicited by the subcutaneous injection of 1 mg/Kg body weight morphine, and it enhanced by 65% the suboptimal analgesic effects of 0.1 mg/Kg morphine. Furthermore, *L. acidophillus*-induced analgesia was significantly inhibited by an intraperitoneally given dose of the cannabinoid receptor 2 (CB2) selective antagonist AM-630, but not by the opioid receptor antagonist naloxone methiodide, thus giving indirect evidence for the physiological role of CB2 in the regulation of splanchnic pain [146].

The combination of *Bifidobacterium longum* and *Lactobacillus helveticus* as a pretreatment in mice subjected to water avoidance stress significantly reduced chronic stress-induced visceral hypersensitivity, in comparison with pretreatment using a single one. Furthermore, the combination of *L. helveticus* and *B. longum* was superior in regulating glucocorticoid negative feedback on the HPA axis [153].

The same authors, having previously reported that *Lactobacillus farciminis* suppresses stress-induced hypersensitivity in response to colorectal distension, evaluated whether this antinociceptive result is related to changes in neuronal activation at spinal and supraspinal sites induced by *L. farciminis* strains. The neuronal activation was assessed by means of Fos protein expression, which is a marker of neuronal activation, rapidly expressed in neurons of the central nervous system in response to somato-cutaneous or splanchnic sensory stimuli. They then assessed whether restraint stress-induced splanchnic hyperalgesia in rats modifies Fos protein expression provoked via colorectal distension and whether this expression can be modulated by *L. farciminis*. After colorectal distension or restraint stress, Fos expression was found to be increased in the sacral spinal cord, in the nucleus tractus solitarius, in the hypothalamus paraventricular nucleus, and in the medial nucleus of the amygdala. When both stimuli were applied, Fos was overexpressed in the sacral spinal cord section, in the paraventricular nucleus, and in the medial nucleus of the amygdala, but not in the nucleus tractus solitarius. *L. farciminis* pretreatment largely reduced the number of Fos-positive cells in all these areas, suggesting that its antinociceptive effect is the result of the reduction in the activation of sensory neurons in the spinal and supraspinal levels due to stress [154,155].

The implication of probiota in the modulation of the gut–brain axis and the HPA axis was further documented after an oral administration of *L. plantarum PS128* in rats for a 14 d period. *L. plantarum PS128* was found to inhibit the 5-hydroxytryptophan-induced visceral hypersensitivity during colorectal distension, the effect being followed by decreased serum corticosterone, decreased neurotransmitter protein (substance P, CGRP, BDNF, and NGF) in the spinal cord, and increased glucocorticoid receptor and decreased mineralocorticoid receptor in the amygdala. These findings suggest that *L. plantarum PS128* decreased splanchnic hypersensitivity through modulating the gut–brain axis and the HPA axis [156].

*Faecalibacterium prausnitzii* is an extremely oxygen-sensitive commensal butyrate-producer bacterium, populating the most anaerobic parts of the GI tract of mammals [157]. In a neonatal maternal separation-induced stress model, a decrease in *Faecalibacterium prausnitzii* was confirmed. This is also evidenced in several intestinal disorders, including that of colon anastomosis leakage after colon surgery, with *F. prausnitzii* being generally recognized as a biomarker of intestinal health [157,158,159]. In two experimental models of chronic (neonatal maternal separation) and acute stress (partial restraint stress), colorectal distension was applied for colonic hypersensitivity induction; then, the *F. prausnitzii A2-165* strain or its supernatant were tested [157]. Both demonstrated anti-inflammatory properties, as also seen with others; however, only *F. prausnitzii* decreased colonic sensitivity and exhibited curative antinociceptive properties in response to a colorectal distension [157,160,161]. The authors suggest that the *F. prausnitzii*-induced antinociceptive properties are possibly correlated to intestinal epithelial barrier enhancement, since others support the opinion that it can modulate tight junctions in animal models of low-grade or of acute inflammation [157,162,163].

One of the fundamental receptors responsible for pain perception in the intestine is the transient receptor potential vanilloid 1 (TRPV1), which is a member of the vanilloid receptor family [164,165,166]. It is expressed in spinal and vagal primary afferent neurons and activated by capsaicin, noxious heat, acidosis, depolarization, and endovanilloids [164,166,167]. In a distension-dependent gut pain model, the effectiveness of *Lactobacillus reuteri DSM 17938* was tested. In a dose-dependent manner, *L. reuteri DSM 17938* was found to reduce the jejunal spinal nerve firing evoked by distension; 80% of this reduction was inhibited by the TRPV1 channel antagonist, which mediates nociceptive signals [168]. In order to further confirm the effect of *L. reuteri* on TRPV1, the authors used a murine jejunal mesenteric nerve bundle model in which capsaicin was applied on serosa; capsaicin induces intracellular calcium production in dorsal root ganglion (DRG) neurons but *L. reuteri DSM 17938* inhibits it, as assessed by means of Ca2+ fluorescence intensity [168]. Furthermore, the *L. reuteri DSM* antinociceptive effects, when it was given via gavage, were tested on gastric distension in rats; *L. reuteri DSM* as a pretreatment was found to inhibit bradycardia induction after painful gastric distension [168]. Finally, it was recently found that both isoflurane inhalation and ketamine intravenously affect acetylcholine-activated TRPC4 channels, which considerably inhibit the muscarinic cation current in ileal myocytes, even when G proteins are activated directly by intracellular GTPγS, i.e., through bypassing muscarinic receptors; thus, they are seriously implicated in anesthesia-induced postoperative ileus [169,170].

*Trichinella spiralis* infection-induced muscle hypercontractility in mice was attenuated after *Lactobacillus paracasei* treatment, but not after *Lactobacillus johnsonii*, *Bifidobacterium lactis*, or *Bifidobacterium longum* treatment from days 10 to 21 post-infection. This finding was related to a decrease in the T-helper 2 response triggered by *T. spiralis* and in transforming growth factor-1, cyclooxygenase-2, and prostaglandin E2 levels in muscles. Based on this experiment, the authors conclude that muscle hypercontractility attenuation is a strain-dependent effect, which follows both immunologic response to infection and a direct effect of *L. paracasei* on muscles [171]. Similarly, acute ex vivo exposure of colonic mucosa to *Lactobacillus rhamnosus GG* resulted in a significant (almost 70%) impairment in smooth muscle cell contraction, the effect being attributed to the reduced contractile response to acetylcholine [172].

Using the same colorectal distension-dependent gut pain model as above, the effectiveness of *Lactobacillus rhamnosus JB-1* was also tested; although its visceral antinociceptive activity was confirmed, the nociceptive signals were not found to be mediated by the specific TRPV1 channel antagonist [168]. It had been previously found in rats that *L. rhamnosus JB-1*, as well as *L. reuteri ATCC23172*, later recognized as being *L. rhamnosus*, and *L. plantarum NCIMB 826 [WT]* inhibited pain perception after even the maximum colorectal distension pressure of 80 mmHg, by altering signaling in DRG fibers [173,174]. Similarly, *L. reuteri* treatment was found to inhibit the mechanosensitive response to gastric distension, but the *L. plantarum NCIMB 826* treatment was not [174].

The probiotics *Bifidobacterium infantis 35624*, *Lactobacillus salivarius UCC4331*, or *Bifidobacterium breve UCC2003* were given via gavage for a 14-day period to two strains of visceral normo-sensitive and visceral hypersensitive rats exposed to a novel stress and then to colorectal distension. Their nociceptive responses were analyzed by recording visceral pain behavior. Only *B. infantis 35624* was found to reduce the total number of pain behaviors in the open field assessment, while significantly increasing the threshold pressure of the first pain behavior [175] (Table 2).

#### 4.2.2. Clinical Studies

Based on previous findings [146] on epithelial cell culture and in mice, Ringel-Kulka et al. [176] enrolled 20 women experiencing mild to moderate abdominal pain to receive either *L. acidophilus NCFM* or this in combination with *Bifidobacterium lactis Bi-07* for 21 days. *L. acidophilus* alone, but not with *Bifidobacterium*, induced colonic opioid receptor μ- (MOR) mRNA and protein expression. In contrast, cannabinoid receptor (CNR2) mRNA expression was decreased, as seen in colonic biopsies obtained before and at the end of treatment. Both treatment groups trended towards improvement in symptoms, but not significantly [176].

Intrarectally given butyrate enemas effected a reduction in pain perception and discomfort when given to healthy volunteers [177]. Based on this knowledge, *Roseburia hominis*, a species of the *Lachnospiraceae* family, well known to consume lactate and acetate to synthesize butyrate via different pathways, was administered orally in rats subjected to water avoidance stress [178].

The probiotic strain *Lactobacillus reuteri DSM 17938*, given orally in a dose of 10^8^ CFU for 21 days, was reported to be effective in improving symptoms of infantile colic and thus reducing the crying time [179]. Previous research had revealed that the gut bacteria in colicky infants varies in relation to healthy ones, exhibiting a reduction in bacterial richness and specifically in *Lactobacillus* and *Bifidobacterium* genera, while Gram-negative bacteria are increased [180,181]. Newer research also reveals that 30-day *Lactobacillus reuteri DSM 17938* treatment significantly alters the mRNA levels of the transcription factors retinoid-related orphan receptor-γ (RORγ) and forkhead box P3 (FOXP3) in the peripheral blood, which modulate T-cell responses to gut microbes: it increases the FOXP3 concentration, thus resulting in a decreased RORγ/FOXP3 ratio. In parallel, it increases the percentage of *Lactobacillus* in feces and decreases that of calprotectin [182].

There is also evidence in irritable bowel syndrome patients, in which *B. infantis 35624* was found to alleviate symptoms of pain/discomfort, bloating/distension, and bowel movement difficulty [183,184]. “In a double blind, placebo-controlled study, 214 irritable bowel syndrome patients, fulfilling the Rome Ⅲ criteria, were randomized to receive a capsule containing 10 billion cfu of *L. plantarum* 299v (DSM 9843) or placebo, daily for 4 weeks. The primary endpoint was the improvement of the frequency of abdominal pain episodes. After conclusion of the treatment, both daily frequency of pain as well as pain and bloating severity were found significantly decreased in probiotic treated participants.” [185].

Similarly, in a preclinical study, 13 patients suffering from irritable bowel syndrome were subjected to fecal microbial transfer from one unrelated healthy donor. Thirteen weeks thereafter, patient fecal microbiota profiles clustered closely or distantly to the donor, with the former group showing a more sustained reduction in pain intensity, but not pain frequency, in relation to the latter. The patients who experienced less pain were found having microbial samples that clustered closely to the donor and being significantly enriched with *Akkermansia muciniphila.* Although *A. muciniphila* belongs to the “new generation” of probiotics, which are less studied, the authors hypothesize that SCFAs, end products of mucus degradation by the microbe, may modulate visceral nociception [54] (Table 3).

## 5. Discussion-Conclusions

Pain, the unpleasant sensory and emotional experience associated with actual or potential tissue damage, remains a crucial point in the treatment of the postoperative patient. And this is not only due to the stress and psychological state of the just-operated patient, but also to the double origin of this pain: the pain of the surgical trauma on the abdominal wall, which is purely of an inflammatory etiology, and the visceral pain, which is due to overdistension of the intestine up to its mobilization from the paralytic ileus, resulting from anesthesia. Postoperative pain is exacerbated by the release of proinflammatory cytokines, mainly IL-1β, IL-6, and TNF-α, not only locally but in the spinal dorsal horn tissues, which are further involved in the pain mechanism. It is documented that inflammation reduces the pain threshold of nociceptors and increases the individual pain response.

It would be of interest to identify in the near future (i) whether there is a more specific causal relationship between microbiota changes and postoperative pain; (ii) whether microbiota alterations vary based on the nature or severity of pain; and (iii) whether pain further accelerates microbiota changes in the host.

**Figure 3:**
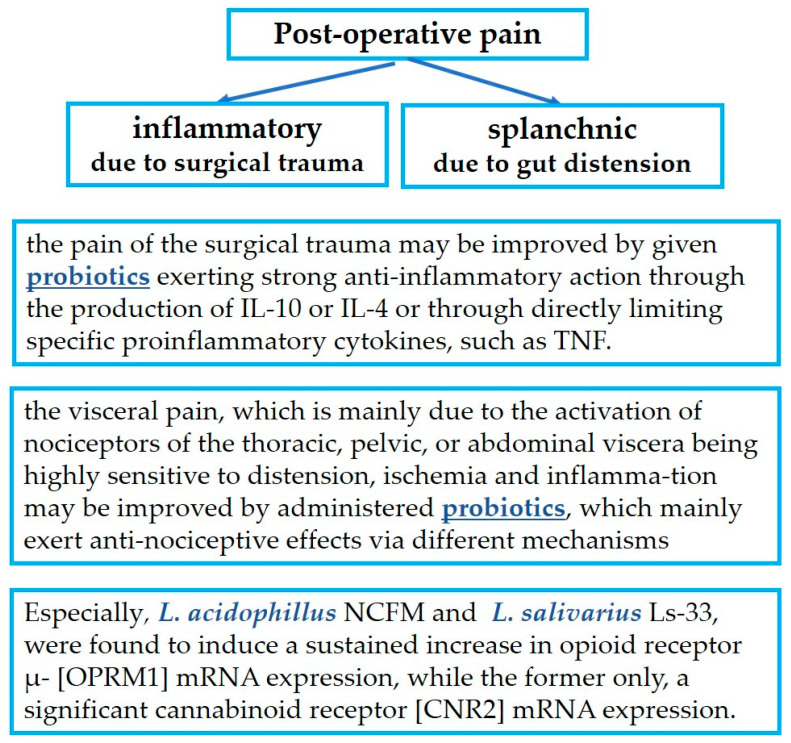
Probiotics in postoperative pain management.

Since pain is linked with alterations in the gut microbiome, and the overall process of surgery is known to alter this microbiome, there has been considerable research towards the treatment of postoperative pain with probiotics. See Figure 3.

Thus, today we know that

- The pain of surgical trauma on the abdominal wall which is of an inflammatory etiology may be improved by giving probiotics that exert strong anti-inflammatory action through the production of IL-10 or IL-4 or through directly limiting specific proinflammatory cytokines, such as TNF. Such benefits have been recognized after treatments mainly with *Lactiplantibacillus plantarum*, and to a lesser extent with *L. acidophilus LA-5*, *L. rhamnosus GG ATCC 53103* and *UBLR-58*, *L. fermentum SGL10*, *L. brevis GQ4237768*, *SGL 12*, and *CECT7480*, *L. paracasei SGL04*, and *MSMC39*, *B. longum UBBL-64* and *Reuter*, and *L. casei Shirota.* Additionally, *Lactobacillus* spp., *B. dentium*, and *Bifidobacterium* spp. are able to modify pain signaling by producing GABA, the most important inhibitory neurotransmitter.
- Visceral pain, which is mainly due to the activation of nociceptors of the thoracic, pelvic, or abdominal organs that are extremely sensitive to distension, tissue ischemia, and inflammation, may be improved by administered probiotics, which mainly exert antinociceptive effects via different mechanisms: *L. plantarum PS128*, *L. acidophilus NCFM*, *L. rhamnosus GG ATCC53103*, *L. reuteri DSM 17938*, *L. paracasei*, *B. infantis 35624*, *B. longum* and *L. helveticus* in combination, *Bifidobacterium lactis CNCM I-2494* and *Lactococcus lactis CNCM I-1631* in combination, and the less known *L. farciminis*, *Roseburia hominis*, a species of the butyrate-producing *Lachnospiraceae* family, and *Faecalibacterium prausnitzii.*
- Finally, particular mention must be made of the extraordinary action of *L. acidophillus NCFM* and of *L. salivarius Ls-33*, which induce a sustained increase in opioid receptor μ- (OPRM1) mRNA expression, while only the former also induces significant cannabinoid receptor (CNR2) mRNA expression.

## Figures and Tables

**Table 1:** Probiotics in relation to the inflammation-induced pain of surgical trauma.

Probiotics	Action	Type of Operation
**Experimental Studies**		
*L. plantarum* [131]	↑ IL-10 and TGF1 ↓ TNF and IL-1	Skin wound healing
*L. plantarum MTCC 2621* [132]	↑ IL-10, earlier re-epithelization, reduction in leukocyte infiltration, increased fibroblasts and collagen	Staphylococcus aureus-infected excisional wounds
*Lactiplantibacillus plantarum* UBLP-40 [133,134]	↑ IL-10 and TGF1 ↓ TNF and IL-1 Earlier re-epithelization, reduction in leukocyte infiltration, increased fibroblasts and collagen	Skin wound healing in rats
*L. rhamnosus UBLR-58* *L. acidophilus LA-5* *L. fermentum SGL10* *L. brevis GQ4237768* *L. brevis SGL 12* *L. paracasei SGL 04* *B. longum UBBL-64* [133,134,135]	↑ IL-10 and TGF1 ↓ TNF and IL-1 Earlier re-epithelization, reduction in leukocyte infiltration, increased fibroblasts and collagen Similar mechanisms—less intensity in relation to *L. plantarum*	Skin wound healing in rats
*B. longum reuter* [137]	Inhibits capsaicin-induced CGRP release	Ex vivo human explant model
*L. rhamnosus GG ATCC 53103* [140,141]	↑ MAPKs	Epithelial cell culture
*L. acidophilus LA-5* [140,141]	↑ MAPKs	Epithelial cell culture
*Lactobacillus* spp. *B. dentium Bifidobacterium* spp. [142]	Produce GABA through enzymatic decarboxylation of glutamate	Skin wound healing in rats
**Clinical Studies**		
*L. paracasei* MSMC 39 [143]	↓ TNF	Mandibular 3rd molar excision
*L. brevis* CECT7480 *L. plantarum* CECT7481 [144]	↓ Pain, eating difficulty	Mandibular 3rd molar excision
*L. casei Shirota* [145]	↓ Pain related to max inspiration	Single rib fracture

Numbers in parentheses represent reference numbers.

**Table 2:** Probiotics in relation to gut distension-induced visceral pain—experimental studies.

Probiotics	Action	Type of Operation
**Experimental Studies**		
*L. acidophillus NCFM* [146]	Increases μ-opioid receptor and cannabinoid receptor R2 expression	Human epithelial cells Mice and rats
*L. salivarius Ls-33* [143]	Increases μ- opioid receptor expression	Human epithelial cells Mice and rats
*L. acidophillus NCFM* [143]	Pain reduction	Colorectal distension in rats
*Akkermansia muciniphila* [47]	Affects the cannabinoid R1 1 and R2 expression	Caco-2 and Hep-G2 cell lines
*Bifidobacterium lactis CNCM I-2494* [147]	Reduces visceral hypersensitivity and pain	Colorectal distension model
*Lactococcus lactis CNCM I-1631* [147]	Reduces visceral hypersensitivity and pain	Colorectal distension model
*L. rhamnosus Lcr35* [148]	IL-13/Th 17 activation Increases IL-13	Colorectal distension model
*L. rhamnosus GG ATCC53103* [149,150]	Alters neurotransmitters	Visceral hypersensitivity model
*Lactobacillus rhamnosus GG* [151]	Decreases splanchnic sensitivity	Maternal separation plus restraint stress
*Roseburia hominis* [152]	Reduction in visceral pain and hypersensitivity via butyrate	Stress
*L. acidophilus NCFM* [146]	Increases colorectal distension threshold	Visceral hypersensitivity model
*Bifidobacterium longum* [153]	Reduces visceral hypersensitivity Regulates glucocorticoid negative feedback on the HPA axis	Water avoidance stress
*Lactobacillus helveticus* [153]	Reduces visceral hypersensitivity Regulates glucocorticoid negative feedback on the HPA axis	Water avoidance stress
*Lactobacillus farciminis* [154,155]	Fos downregulation Reduce visceral hypersensitivity	Colorectal distension model
*L. plantarum PS128* [156]	Inhibits 5-HTP-induced visceral hypersensitivity Modulates gut–brain HPA axis	Colorectal distension model
*Faecalibacterium prausnitzii A2-165* [157,160,161]	Anti-inflammatory properties Antinociceptive properties	Neonatal maternal separation plus colorectal distension
		Partial restraint stress plus colorectal distension model
*Lactobacillus reuteri DSM 17938* [168]	Reduces the jejunal spinal nerve firing via the TRPV1 channel antagonist	Colorectal distension model
	Inhibits capsaicin-induced intracellular calcium in DRGs	Jejunal mesenteric nerve bundles
*Lactobacillus reuteri DSM 17938* [168]	Inhibits bradycardia induced after gastric distension	Gastric distension in rats
*Lactobacillus paracasei* [171]	Decreases T-helper 2 response Decreases TGF-1, COX-2, and PGE2 levels in muscles	Muscle hypercontractility induced by *Trichinella spiralis* infection in mice
*Lactobacillus rhamnosus GG* [172]	Reduce smooth muscle cell contraction via acetylcholine	Ex vivo colonic mucosa
*L.rhamnosus JB-1* [173,174]	Inhibits pain perception by altering signaling in DRG fibers	Colorectal distension model
*L. reuteri* [174]	Inhibits the mechanosensitive response	Gastric distension
*Bifidobacterium infantis 35624* [175]	Reduces the pain behavior and increases the threshold pressure	Stress plus colorectal distension model

Numbers in parentheses represent reference numbers.

**Table 3:** Probiotics in relation to gut distension-induced visceral pain—clinical studies.

Probiotics	Action	Type of Operation
**Clinical studies**		
*L. acidophilus NCFM* [176]	Increases μ-opioid receptor and cannabinoid receptor R2 expression	Females, mild to mode-rate abdominal pain
Butyrate enemas [177]	Reduction in pain perception and discomfort	Healthy volunteers
*Lactobacillus reuteri DSM 17938* [179]	Reduces the crying time	Infantile colic
*Lactobacillus reuteri* DSM 17938 [182]	Increases the FOXP3 concentration and decreases RORγ/FOXP3 ratio Modulates T-cell response to microbes	30 days treatment
*B. infantis 35624* [183,184]	Alleviates symptoms of pain/discomfort, bloating/distension, and bowel movement difficulty	Irritable bowel syndrome patients
*L. plantarum 299v* (DSM 9843) [185]	Reduction in pain episodes Reduction in pain and bloating severity	Irritable bowel syndrome patients
*Akkermansia muciniphila* [54]	Patients with fecal microbiota profiles clustered closely to the donor’s experience less pain, and feces found enriched with *A. muciniphila*, like donor’s	Fecal microbial transfer Preclinical study Irritable bowel syndrome

Numbers in parentheses represent reference numbers.

## Data Availability

All data in this article will be made available on reasonable demand.
